# Coffee and caffeine consumption and risk of renal cell carcinoma: A Mendelian randomization study

**DOI:** 10.3389/fnut.2022.898279

**Published:** 2022-08-22

**Authors:** Bing-Hui Li, Si-Yu Yan, Xu-Hui Li, Qiao Huang, Li-Sha Luo, Yun-Yun Wang, Jiao Huang, Ying-Hui Jin, Yong-Bo Wang

**Affiliations:** ^1^Department of Urology, Zhongnan Hospital of Wuhan University, Wuhan, China; ^2^Center for Evidence-Based and Translational Medicine, Zhongnan Hospital of Wuhan University, Wuhan, China; ^3^Department of Geriatrics, Zhongnan Hospital of Wuhan University, Wuhan, China

**Keywords:** renal cell carcinoma, coffee consumption, caffeine consumption, Mendelian randomization, causal effect

## Abstract

**Background:**

The association between coffee and caffeine consumption and the risk of renal cell carcinoma was inconsistent among observational studies, and whether these observed associations were causal remained unclear. Therefore, we performed two-sample Mendelian randomization (MR) study to assess the causal nature of the association.

**Materials and methods:**

In this study, 12 and two independent single nucleotide polymorphisms (SNPs) related to coffee and caffeine consumption at a genome-wide significance level of *p* < 5 × 10^–8^ were used as instrumental variables (IVs), respectively. Summary-level data for renal cell carcinoma were taken from the FinnGen consortium with up to 174,977 individuals, and the International Agency for Research on Cancer (IARC) with 13,230 individuals. We used inverse-variance weighted (IVW) as the main method, followed by the weighted median method, the MR-Egger regression method, and the MR robust adjusted profile score method. Outlier and pleiotropic variants were assessed by the MR Pleiotropy RESidual Sum and Outlier test and MR-Egger regression. We used meta-analysis methods in fixed-effects to combine the estimates from the two sources.

**Results:**

The genetically predicted coffee consumption was not associated with the risk of renal cell carcinoma in the FinnGen consortium, and the relationship was consistent in the IARC consortium. The pooled odds ratio (*OR*) per 50% increase of coffee consumption was 0.752 [95% confidence interval (*CI*), 0.512–1.105; *p* = 0.147]. In addition, complementary analyses that separated the coffee-related SNPs according to their relationship with blood levels of caffeine metabolites (higher, lower, or unrelated) found no relationship with renal cell carcinoma. The results were consistent after excluding eight SNPs due to potential risk factors at genome-wide significance (*p* < 5 × 10^–8^). Moreover, genetically predicted per 80-mg increase in caffeine consumption was not associated with the risk of renal cell carcinoma (pooled *OR* = 0.872, 95% *CI*: 0.676–1.125, *p* = 0.292).

**Conclusion:**

Our MR study provided no convincing evidence for a causal effect between coffee and caffeine consumption and the risk of renal cell carcinoma. The associations for renal cell carcinoma need to be verified in well-powered studies.

## Introduction

The incidence rate of renal cell carcinoma has increased significantly by approximately 1.1% every year in the past few years (1). Compared with 1990, the global incident cases in 2019 were higher by 154.78% for renal cell carcinoma (2). During the 30-year study period, there was an upward trend in the age-standardized mortality rate and the age-standardized disability-adjusted life-years rate for renal cell carcinoma (estimated annual percentage change = 0.35 and 0.12, respectively) (2). Previous studies demonstrated several risk factors for renal cell carcinoma, but the accurate pathogenesis remains unclear (3, 4). Given that renal cell carcinoma is a fatal disease, it is crucial to identify interventions that can reduce the risk of this disease.

Coffee is one of the most popular beverages worldwide. A traditional cup of coffee could contain up to 1,000 bioactive compounds, such as a wide variety of aromatic compounds, antioxidants, and most importantly, caffeine (5). Several researchers have been interested in the relationship between coffee and caffeine consumption and tumor risk, given the anti-inflammatory and antioxidant properties of the beneficial ingredients (6–8). However, the relationship between coffee and caffeine consumption and renal cell carcinoma remains controversial. For instance, certain observational studies suggested that the consumption of coffee and caffeine is a protective factor for renal cell carcinoma (9, 10). Other studies, however, suggested otherwise (11).

As the kidney is an excretory organ, the role of fluid consumption could also be important in the development of renal cell carcinoma. However, observational studies on coffee and caffeine consumption and renal cell carcinoma risk may have several potential limitations. First, the observed acute effects of coffee and caffeine may not reflect their long-term effects, because the body can develop a tolerance to caffeine (12). Second, traditional epidemiological studies on coffee and caffeine intake and renal cell carcinoma risk may be affected by confounding factors (such as smoking or other unhealthy lifestyles) and reverse causation, and early studies that did not fully consider these biases may have produced misleading results (11, 13–16). Even in recent studies that have conducted a more comprehensive correction for potential confounding factors, residual confounding is still a worrying issue (10). Since the available evidence on the association between coffee and caffeine consumption and renal cell carcinoma risk came from traditional observational studies, the conclusions might be biased by reverse causation and residual confounding factors. Therefore, it is still indistinct whether coffee and caffeine intake plays a causal role in the renal cell carcinoma risk.

Randomized controlled trials (RCTs) cannot be conducted due to practice, cost considerations, and ethics issues. Mendelian randomization (MR) analysis could enhance casual inference on the association of exposure and an outcome of interest by employing genetic instrumental variants as instrumental variables (IVs) of exposure factors. The IVs are unlikely to be correlated with confounders associated with exposure and outcome of interest, as they are randomly allocated at the time of pregnancy. In addition, MR analysis reduces reverse causation because allele randomization is occurred before the development of disease.

We performed an MR analysis to evaluate the causal relationship between coffee and caffeine consumption and the risk of renal cell carcinoma.

## Materials and methods

### Study design

**FIGURE 1:**
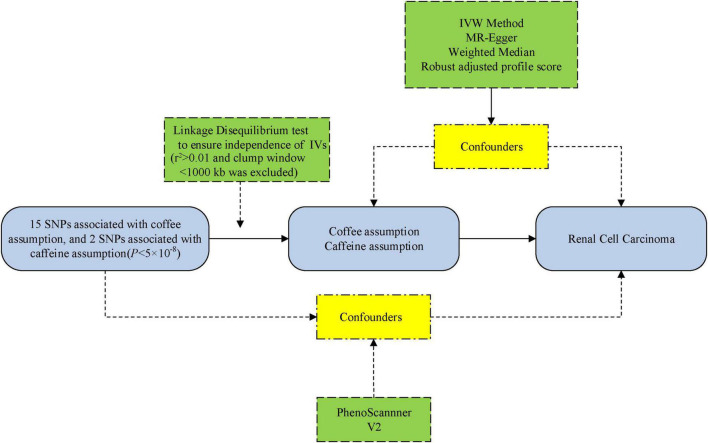
The flowchart of the Mendelian randomization (MR) study. IVs, instrumental variables; SNP, single nucleotide polymorphism; IVW, inverse-variance-weighted.

We performed an MR analysis based on three core assumptions: (1) the IVs are robustly related to the exposure of interest, (2) the IVs are not affected by any confounding factors, (3) and the IVs do not influence the outcome *via* any variable other than the exposure (17). The flowchart of this MR study design is displayed in Figure 1.

### Genetic instrument selection for coffee and caffeine consumption

This study was on the strength of publicly retrievable summary-level data from large-scale genome-wide association studies (GWASs) and consortium that previously obtained informed consent and ethics review board approvals. The single-nucleotide polymorphisms (SNPs) closely related to coffee intake were derived from a meta-analysis of four GWASs (the United Kingdom Biobank and three United States cohorts) (18). The GWASs adjusted for sex, age, total energy, body mass index, and top 20 principal components. In the United Kingdom Biobank (discovery phase), a touch screen questionnaire was applied to collect coffee consumption from all participants at baseline: “How many cups of coffee do you drink each day (including decaffeinated coffee)?” In the United States cohorts (replication phase), a semi-quantitative food frequency questionnaire was used to collect the regular and decaffeinated coffee consumption. The GWASs contained 375,833 individuals of European descent, and the median coffee consumption ranged from 1.1 to 2.5 cups per day. The effect sizes for the SNP-coffee associations were expressed per 1% of increase in coffee consumption in the GWASs. The odds ratio (*OR*) estimates of renal cell carcinoma were scaled to a per 50% of increase in coffee consumption in our study. To fulfill the first MR assumption that IVs are robustly related to the exposure of interest, 15 SNPs that were related to coffee consumption at the level of genome-wide significance (*p* < 5 × 10^–8^) (18) were identified as IVs (Supplementary Table 1). Selected SNPs explained approximately 0.48% phenotypic variance in coffee consumption (18). We calculated the linkage disequilibrium (LD) between the 15 SNPs based on an LD reference panel from 1,000 Genomes of European populations. We excluded SNPs in LD (*r*^2^ > 0.01 and clump window < 1,000 kb) and retained the SNP with the lowest *p*-value. To fulfill the second MR condition that the IVs are not affected by any confounding factors, we evaluated the pleiotropic relationships of the SNPs with potential confounding factors by searching the PhenoScanner V2 website.^1^ The LD of these 15 SNPs was computed utilizing the 1,000 genomes LD European individual panel as a reference population (19). There were 3 of the 15 SNPs excluded due to LD (rs117692895, rs4719497, and rs12699844 in chromosome 7). Therefore, 12 SNPs were implemented as IVs for coffee consumption in the primary analysis. Among the selected 12 SNPs in the primary analysis, the coffee-raising allele has been found to be correlated with higher (one SNP, rs2330783), lower (four SNPs, rs1260326, rs1057868, rs4410790, and rs2472297), and irrelevant to blood levels of caffeine metabolites (five SNPs, rs574367, rs10865548, rs597045, rs1956218, and rs66723169); and for two SNPs (rs34060476 and rs73073176), the relationship was unclear (20, 21). We then excluded eight SNPs due to potential risk factors at genome-wide significance (*p* < 5 × 10^–8^) (Supplementary Tables 2, 3). The rest of the four SNPs were employed as IVs for coffee consumption in the sensitivity analysis.

A total of two SNPs correlated with caffeine consumption (*p* < 5 × 10^–8^) were applied as IVs for caffeine from a meta-analysis of six GWAS (a total of 9,876 people of European descent) (21). A self-reported questionnaire was used to measure caffeine consumption from the consumption of coffee, tea, and cola (22). The summary-level data for SNPs related to caffeine intake (i.e., beta coefficient and standard error [SE]) were obtained. The effect size for the SNP-caffeine association was scaled to an 80-mg raise, which was equal to the caffeine dose of one cup of coffee (22). These two SNPs approximately explained a 1.31% variance for caffeine consumption (Supplementary Table 5).

### Data source for renal cell carcinoma

We acquired summary-level data for genetic association with renal cell carcinoma from the FinnGen consortium and the International Agency for Research on Cancer (IARC) (Supplementary Tables 4, 5). The fifth release of the FinnGen consortium data included a total of 174,977 men and women of Finnish ancestry after the removal of individuals with excess heterozygosity (±4 SD), high genotype missingness (>5%), ambiguous gender, and non-Finnish ancestry (23). All genetic association effect sizes were computed by multivariable logistic regression and adjusted for age, sex, and genetic principal components. In IARC, the dataset of renal cell carcinoma was a gender-specific GWAS meta-analysis comprising two kidney cancer genome-wide scans for women (1,992 cases and 3,095 controls) and men (3,227 cases and 4,916 controls) of European descent (24). The quality control, imputation, and sex-specific association analysis protocols were described in one study (24).

### Statistical analysis

We used the inverse-variance weighted (IVW) method with random effects to assess the relationships for genetically predicted coffee consumption and the IVW method with fixed effects (for analysis with less than three SNPs) to assess the relationships for genetically predicted caffeine consumption (25). Several other analyses were carried out, including the weighted median (WM) method (26), the MR-Egger regression method (27), and the MR robust adjusted profile score (MR-RAPS) method (28). The IVW method uses coefficients and standard errors uniting with risk factors and regresses the results of each genetic variation in turn (25). The WM combines data from multiple genetic instruments for the consistency analysis by computing a single weighted median estimator (26). The MR-Egger method allows each IV to exhibit pleiotropy effects and is consistent if the instrument strength is independent of these pleiotropic effects (27). The MR-RAPS is more robust to weak instrument bias (28). The MR pleiotropy residual sum and outlier (MR-PRESSO) method was applied to identify potential outlier SNPs (29). We used Cochran’s *Q* statistic to test the heterogeneity of the IVW and MR-Egger methods for the causal estimates of individual SNPs. To examine the third MR assumption that the IVs do not influence the outcome *via* any variable other than the exposure, we calculated the intercept and 95% confidence interval (*CI*) of the MR-Egger regression line for testing horizontal pleiotropy (27). In the complementary analysis, we grouped the coffee-related SNPs according to their relationship with blood levels of caffeine metabolites (20, 21) and separately conducted MR analyses for each group of SNPs by the IVW method. We computed the F-statistic to evaluate the strength of the instruments (30). The statistical analysis was implemented using R (version 4.0.2), through MR-PRESSO and TwoSample MR packages. All statistical tests were two-sided, and the evidence of association was cutoff at a prespecified *p*-value below 0.05 in the final MR analyses.

## Results

### F-statistic and outlier detection

The F-statistic for coffee and caffeine consumption was 159 and 67, respectively, suggesting that the selected SNPs were adequate in strength as IVs for both coffee and caffeine consumption. In addition, no outlier SNPs were found in the MR-PRESSO in all primary and sensitivity analyses.

### Causal relationships between coffee and caffeine consumption and renal cell carcinoma

**FIGURE 2:**
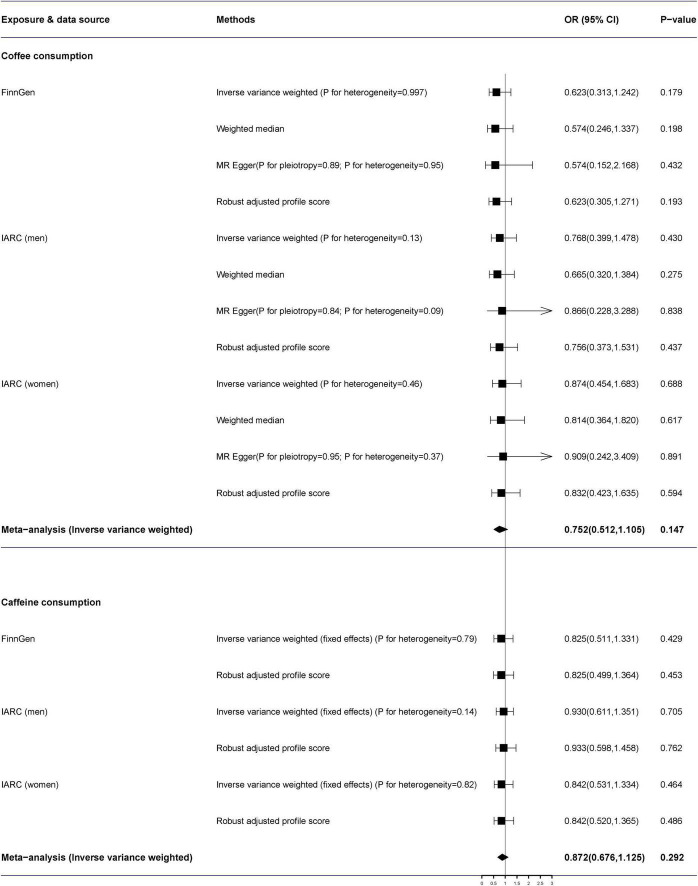
The association of genetically predicted coffee and caffeine consumption with renal cell carcinoma. Pooled estimates were combined using the fixed-effects meta-analysis methods. *ORs* for renal cell carcinoma were scaled to a genetically predicted 50% of increase in coffee consumption and an 80-mg increase in caffeine consumption. FinnGen, FinnGen Consortium; IACC, the International Agency for Research on Cancer; *OR*, odds ratio; CI, confidence interval.

**FIGURE 3:**
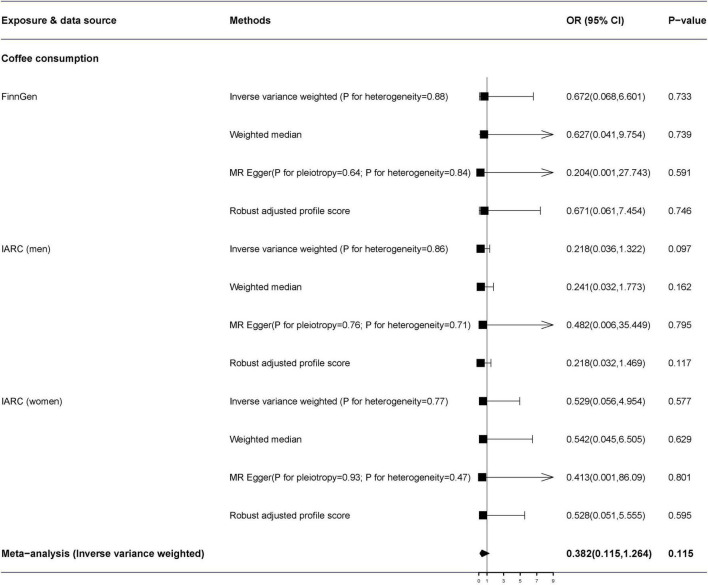
Results of sensitivity analyses association of genetically predicted coffee consumption with renal cell carcinoma. Estimates were obtained from the inverse-variance weighted methods and combined using the fixed-effects meta-analysis methods. *ORs* for renal cell carcinoma were scaled to a genetically predicted 50% of increase in coffee consumption. FinnGen, FinnGen Consortium; IACC, the International Agency for Research on Cancer; *OR*, odds ratio; CI, confidence interval.

The MR-Egger analysis identified no directional pleiotropy (all *p* > 0.05) (Figure 2). Additionally, we detected no heterogeneity by the IVW and MR-Egger regression. In the primary analysis, genetically predicted coffee consumption was not correlated with renal cell carcinoma in the FinnGen consortium study (*OR* = 0.623, 95% *CI*: 0.313–1.242) by the IVW method in the random-effects model (Figure 2). In addition, there was no effect of coffee on renal cell carcinoma in IARC consortium [men (*OR* = 0.768, 95% *CI*: 0.399–1.478, *p* = 0.430) and women (*OR* = 0.874, 95% *CI*: 0.454–1.683, *p* = 0.688)]. The combined *OR* of per genetically predicted 50% increase of coffee consumption was 0.752 (95% *CI*: 0.512–1.105, *p* = 0.147). Moreover, there was no obvious difference between the results of the sensitivity analysis and the primary analysis (Figure 3).

Complementary analyses that distinguished the genetically predicted coffee-related SNPs according to their correlation with caffeine metabolites blood levels (higher, lower, or unrelated) showed no significant difference (Supplementary Table 6).

The per 80-mg increase in caffeine consumption was also not associated with a risk of renal cell carcinoma (combined *OR* = 0.872, 95% *CI*: 0.676–1.125, *p* = 0.292) (Figure 2).

## Discussion

Our study did not find a relationship between genetically predicted coffee and caffeine consumption and the risk of renal cell carcinoma. No outlier SNPs or pleiotropy were detected. Sensitivity analyses showed consistent results. In addition, coffee related genetically predicted SNPs that are correlated with higher or lower levels of caffeine metabolites in the blood, reflecting slower or faster caffeine metabolism, respectively, showed no significant difference.

Previous studies have reported inconsistent results on the association between coffee or caffeine consumption and the risk of renal cell carcinoma. A recent meta-analysis including 16 case-control and 6 cohort studies identified no significant relationship between coffee consumption and the risk of renal cell carcinoma in men and women, with a relative risk of 0.87 (95% *CI*: 0.72–1.04) and 1.15 (95% *CI*: 0.85–1.55), respectively (13). In addition, a meta-analysis including 13 cohorts found insignificant association for coffee consumption and kidney cancer in a dose-response analysis (14). However, a meta-analysis of 10 cohort studies detected a pooled relative risk of 0.85 (95% *CI*: 0.76–0.96) comparing the risk of renal cancer between the highest and the lowest category of coffee consumption, and this inverse relationship still remained among studies adjusting for body mass index (BMI) and smoking (16). Meanwhile, a case-control study with 669 renal cell carcinoma cases and 1,001 matched controls found an inverse correlation between caffeinated coffee intake and renal cell carcinoma risk (*OR* = 0.74, 95% *CI*: 0.57–0.99) in comparison with no coffee consumption, whereas there was a trend toward increased risk of renal cell carcinoma for decaffeinated coffee consumption (*OR* = 1.47, 95% *CI*: 0.98–2.19) (15). Generally, the cancer-coffee relationship is strongly modified by smoking, and this phenomenon was observed not only for renal cell carcinoma only but generally for cancer (31). A recently published large prospective cohort study with a total of 420,118 participants found an inverse relationship between coffee consumption and renal cell carcinoma after adjustment for several risk factors, with a 20% reduction in the risk of developing renal cell carcinoma during a 16-year follow-up for those who drank ≥ 2 cups of coffee per day over 16 years of follow-up (10). The inverse relationship was detected among non-smokers but not ever-smokers.

Whereas, those observational studies might be limited by their research design and could not rule out residual confounding (such as healthy lifestyle factors and dietary components) and reverse causality that influenced the results. Besides, the measurement of long-term coffee and caffeine consumption in observational epidemiological studies may be inexact.

This is the first two-sample MR method to assess the potential relationship between coffee and caffeine consumption and the risk of renal cell carcinoma. The two-sample MR method is not subject to interferences by confounding factors, such as lifestyle and social environment and reverse causation by using IVs as a proxy for lifetime coffee and caffeine consumption. In this study, LD analysis was used to exclude three SNPs of coffee IV, and the MR-Egger intercept test identified that the selected 12 SNPs did not have pleiotropic properties, which also increased the credibility of our study. In our study, the IVs for coffee consumption may be related to other risk factors for renal cell carcinoma, and thus the relationship between the genetic variants and renal cell carcinoma may be susceptible to confounding by these factors. The four SNPs in the sensitivity analysis were not related to other traits, indicating that this association was not affected by confounding factors between coffee-related SNPs and renal cell carcinoma. Moreover, we assessed these relationships in two independent populations, and the consistent results ensured the stability of the research findings.

There were several limitations. One restriction is that the current MR analysis assumes a linear relationship between exposure and the outcome of interest. If there is a non-linear relationship or threshold effect between habitual coffee and caffeine consumption and renal cell carcinoma, we cannot detect this association. Therefore, our null detections may not rule out a possible beneficial effect of moderate but not massive coffee and caffeine consumption on renal cell carcinoma. In addition, in our complementary analyses, we might not have enough power to detect the weak relationships between various levels of caffeine metabolites and the risk of renal cell carcinoma. Therefore, further research on the causal relationship between caffeine metabolite exposure and the risk of renal cell carcinoma is needed to verify our findings. Beyond that, the GWAS for coffee consumption was based on regular and decaffeinated coffee consumption. However, we added the effects of caffeine consumption on the risk of renal cell carcinoma, and the results were consistent with coffee consumption. Moreover, it is worth mentioning that the population of our study is limited to European individuals; this conclusion may not be directly applicable to other populations. Furthermore, there is an urgent need for more studies with larger sample sizes to explore the relationship among different coffee bean types, roasting procedures, brewing methods, and renal cell carcinoma.

## Conclusion

Our MR study provided no convincing evidence for a causal effect between coffee and caffeine consumption and the risk of renal cell carcinoma. Further longitudinal and experimental studies are still demanded to authenticate our results.

## Data availability statement

The original contributions presented in this study are included in the article/Supplementary material, further inquiries can be directed to the corresponding authors.

## Author contributions

Y-BW, Y-HJ, and B-HL contributed to the protocol development, data collection, and statistical analysis. B-HL, S-YY, X-HL, and QH drafted the manuscript. L-SL, Y-YW, and JH supervised the method and visualized the results. All authors contributed to manuscript revision, read, and approved the submitted version.

## Footnotes

1
http://www.phenoscanner.medschl.cam.ac.uk/

## References

[1] Palumbo C Pecoraro A Knipper S Rosiello G Luzzago S Deuker M Contemporary age-adjusted incidence and mortality rates of renal cell carcinoma: analysis according to gender, race, stage, grade, and histology. *Eur Urol Focus.* (2021) 7:644–52. 10.1016/j.euf.2020.05.003 32456993

[2] Zi H He SH Leng XY Xu XF Huang Q Weng H Global, regional, and national burden of kidney, bladder, and prostate cancers and their attributable risk factors, 1990-2019. *Mil Med Res.* (2021) 8:60. 10.1186/s40779-021-00354-z 34819142 PMC8611255

[3] Capitanio U Bensalah K Bex A Boorjian SA Bray F Coleman J Epidemiology of renal cell carcinoma. *Eur Urol.* (2019) 75:74–84. 10.1016/j.eururo.2018.08.036 30243799 PMC8397918

[4] Yuan S Fang C Leng WD Wu L Li BH Wang XH Oral microbiota in the oral-genitourinary axis: identifying periodontitis as a potential risk of genitourinary cancers. *Mil Med Res.* (2021) 8:54. 10.1186/s40779-021-00344-1 34588004 PMC8480014

[5] Butt MS Sultan MT. Coffee and its consumption: benefits and risks. *Crit Rev Food Sci Nutr.* (2011) 51:363–73. 10.1080/10408390903586412 21432699

[6] Freedman ND Park Y Abnet CC Hollenbeck AR Sinha R. Association of coffee drinking with total and cause-specific mortality. *N Engl J Med.* (2012) 366:1891–904. 10.1056/NEJMoa1112010 22591295 PMC3439152

[7] Poole R Kennedy OJ Roderick P Fallowfield JA Hayes PC Parkes J. Coffee consumption and health: umbrella review of meta-analyses of multiple health outcomes. *BMJ.* (2017) 359:j5024. 10.1136/bmj.j5024 29167102 PMC5696634

[8] Ludwig IA Clifford MN Lean ME Ashihara H Crozier A. Coffee: biochemistry and potential impact on health. *Food Funct.* (2014) 5:1695–717. 10.1039/C4FO00042K 24671262

[9] Nilsson LM Johansson I Lenner P Lindahl B Van Guelpen B. Consumption of filtered and boiled coffee and the risk of incident cancer: a prospective cohort study. *Cancer Causes Control.* (2010) 21:1533–44. 10.1007/s10552-010-9582-x 20512657

[10] Rhee J Loftfield E Freedman ND Liao LM Sinha R Purdue MP. Coffee consumption and risk of renal cell carcinoma in the NIH-AARP Diet and Health Study. *Int J Epidemiol.* (2021) 50:1473–81. 10.1093/ije/dyab011 33624757 PMC8783596

[11] Hu J Mao Y DesMeules M Csizmadi I Friedenreich C Mery L. Total fluid and specific beverage intake and risk of renal cell carcinoma in Canada. *Cancer Epidemiol.* (2009) 33:355–62. 10.1016/j.canep.2009.10.004 19896918

[12] Beaumont R Cordery P Funnell M Mears S James L Watson P. Chronic ingestion of a low dose of caffeine induces tolerance to the performance benefits of caffeine. *J Sports Sci.* (2017) 35:1920–7. 10.1080/02640414.2016.1241421 27762662

[13] Wijarnpreecha K Thongprayoon C Thamcharoen N Panjawatanan P Cheungpasitporn W. Association between coffee consumption and risk of renal cell carcinoma: a meta-analysis. *Intern Med J.* (2017) 47:1422–32. 10.1111/imj.13621 28892303

[14] Huang TB Guo ZF Zhang XL Zhang XP Liu H Geng J Coffee consumption and urologic cancer risk: a meta-analysis of cohort studies. *Int Urol Nephrol.* (2014) 46:1481–93. 10.1007/s11255-014-0699-9 24677003

[15] Antwi SO Eckel-Passow JE Diehl ND Serie DJ Custer KM Arnold ML Coffee consumption and risk of renal cell carcinoma. *Cancer Causes Control.* (2017) 28:857–66. 10.1007/s10552-017-0913-z 28647866 PMC5782814

[16] Rhee J Lim RK Purdue MP. Coffee consumption and risk of renal cancer: a meta-analysis of cohort evidence. *Cancer Causes Control.* (2022) 33:101–8. 10.1007/s10552-021-01506-1 34677742 PMC8738141

[17] Hemani G Bowden J Davey Smith G. Evaluating the potential role of pleiotropy in Mendelian randomization studies. *Hum Mol Genet.* (2018) 27:R195–208. 10.1093/hmg/ddy163 29771313 PMC6061876

[18] Zhong VW Kuang A Danning RD Kraft P van Dam RM Chasman DI A genome-wide association study of bitter and sweet beverage consumption. *Hum Mol Genet.* (2019) 28:2449–57. 10.1093/hmg/ddz061 31046077 PMC6606847

[19] Clarke L Zheng-Bradley X Smith R Kulesha E Xiao C Toneva I The 1000 genomes project: data management and community access. *Nat Methods.* (2012) 9:459–62. 10.1038/nmeth.1974 22543379 PMC3340611

[20] Cornelis MC Munafo MR. Mendelian randomization studies of coffee and caffeine consumption. *Nutrients.* (2018) 10:1343. 10.3390/nu10101343 30241358 PMC6213346

[21] Cornelis MC Kacprowski T Menni C Gustafsson S Pivin E Adamski J Genome-wide association study of caffeine metabolites provides new insights to caffeine metabolism and dietary caffeine-consumption behavior. *Hum Mol Genet.* (2016) 25:5472–82. 10.1093/hmg/ddw334 27702941

[22] McMahon G Taylor AE Davey Smith G Munafò MR. Phenotype refinement strengthens the association of AHR and CYP1A1 genotype with caffeine consumption. *PLoS One.* (2014) 9:e103448. 10.1371/journal.pone.0103448 25075865 PMC4116211

[23] FinnGen consortium. *FinnGen Documentation of R5 Release.* (2021). Available online at: https://finngen.gitbook.io/documentation/ (accessed September 1, 2020).

[24] Laskar RS Muller DC Li P Machiela MJ Ye Y Gaborieau V Sex specific associations in genome wide association analysis of renal cell carcinoma. *Eur J Hum Genet.* (2019) 27:1589–98. 31231134 10.1038/s41431-019-0455-9 PMC6777615

[25] Burgess S Butterworth A Thompson SG. Mendelian randomization analysis with multiple genetic variants using summarized data. *Genet Epidemiol.* (2013) 37:658–65. 10.1002/gepi.21758 24114802 PMC4377079

[26] Bowden J Davey Smith G Haycock PC Burgess S. Consistent estimation in Mendelian randomization with some invalid instruments using a weighted median estimator. *Genet Epidemiol.* (2016) 40:304–14. 10.1002/gepi.21965 27061298 PMC4849733

[27] Bowden J Davey Smith G Burgess S. Mendelian randomization with invalid instruments: effect estimation and bias detection through egger regression. *Int J Epidemiol.* (2015) 44:512–25. 10.1093/ije/dyv080 26050253 PMC4469799

[28] Zhao Q Chen Y Wang J Small DS. Powerful three-sample genome-wide design and robust statistical inference in summary-data Mendelian randomization. *Int J Epidemiol.* (2019) 48:1478–92. 10.1093/ije/dyz142 31298269

[29] Verbanck M Chen CY Neale B Do R. Detection of widespread horizontal pleiotropy in causal relationships inferred from Mendelian randomization between complex traits and diseases. *Nat Genet.* (2018) 50:693–8. 10.1038/s41588-018-0099-7 29686387 PMC6083837

[30] Pierce BL Ahsan H Vanderweele TJ. Power and instrument strength requirements for Mendelian randomization studies using multiple genetic variants. *Int J Epidemiol.* (2011) 40:740–52. 10.1093/ije/dyq151 20813862 PMC3147064

[31] Grosso G Micek A Godos J Sciacca S Pajak A Martínez-González MA Coffee consumption and risk of all-cause, cardiovascular, and cancer mortality in smokers and non-smokers: a dose-response meta-analysis. *Eur J Epidemiol.* (2016) 31:1191–205. 10.1007/s10654-016-0202-2 27699514

