# Supplementary material for: Coffee and caffeine consumption and risk of renal cell carcinoma: A Mendelian randomization study
Source: Front Nutr. 2022 Aug 22;9:898279. doi: 10.3389/fnut.2022.898279 (PMC9441794; doi:10.3389/fnut.2022.898279)
Supplement: Supplementary file 1 [file Data_Sheet_1.docx]

**Supplementary Materials**

Supplementary Table 1. Details of studies and datasets used for analyses

Supplementary Table 2. Characteristics of the single-nucleotide polymorphisms associated with coffee consumption

Supplementary Table 3. Potential secondary phenotypes of the genetic variants used for coffee

Supplementary Table 4. Associations of single nucleotide polymorphisms for coffee consumption and renal cell carcinoma

Supplementary Table 5. Associations of single nucleotide polymorphisms for caffeine consumption and renal cell carcinoma

Supplementary Table 6. Estimated causal effect of coffee consumption on renal cell carcinoma with caffeine metabolite levels by the inverse-variance weighted method

Supplementary Table 1. Details of studies and datasets used for analyses

| Exposure or outcome | Study or consortium | Participants | Ethnicity | Web source |
| --- | --- | --- | --- | --- |
| Coffee consumption | Zhong VW | 375,833 | European | https://academic.oup.com/hmg/article/28/14/2449/5424254 |
| Caffeine consumption | Cornelis MC | 9,876 | European | https://academic.oup.com/hmg/article/25/24/5472/2581117 |
| Renal cell carcinoma | FinnGen | 971/174,006 | European | <https://storage.googleapis.com/finngen-public-data-r5/summary_stats/> |
| Renal cell carcinoma in men | International Agency for Research on Cancer | 3,227/4,916 | European | <https://www.nature.com/articles/s41431-019-0455-9> |
| Renal cell carcinoma in women | International Agency for Research on Cancer | 1,992/3,095 | European | <https://www.nature.com/articles/s41431-019-0455-9> |

Supplementary Table 2. Characteristics of the single-nucleotide polymorphisms associated with coffee consumption

| Exposure | Chromosome | SNP | Included in the primary MR analysis | Included in the sensitivity MR analysis | Association with caffeine metabolite levels |
| --- | --- | --- | --- | --- | --- |
| Coffee | 1 | rs574367 | Yes | No, Horizontal pleiotropy | Unrelated |
| Coffee | 2 | rs10865548 | Yes | No, Horizontal pleiotropy | Unrelated |
| Coffee | 2 | rs1260326 | Yes | No, Horizontal pleiotropy | Lower blood levels of caffeine metabolites |
| Coffee | 7 | rs1057868 | Yes | No, Horizontal pleiotropy | Lower blood levels of caffeine metabolites |
| Coffee | 7 | rs117692895 | No, Linkage disequilibrium | No, Linkage disequilibrium | - |
| Coffee | 7 | rs12699844 | No, Linkage disequilibrium | No, Linkage disequilibrium | - |
| Coffee | 7 | rs34060476 | Yes | No, Horizontal pleiotropy | Unknown |
| Coffee | 7 | rs4410790 | Yes | No, Horizontal pleiotropy | Lower blood levels of caffeine metabolites |
| Coffee | 7 | rs4719497 | No, Linkage disequilibrium | No, Linkage disequilibrium | - |
| Coffee | 7 | rs73073176 | Yes | Yes | Unknown |
| Coffee | 11 | rs597045 | Yes | Yes | Unrelated |
| Coffee | 14 | rs1956218 | Yes | Yes | Unrelated |
| Coffee | 15 | rs2472297 | Yes | No, Horizontal pleiotropy | Lower blood levels of caffeine metabolites |
| Coffee | 18 | rs66723169 | Yes | No, Horizontal pleiotropy | Unrelated |
| Coffee | 22 | rs2330783 | Yes | Yes | Higher blood levels of caffeine metabolites |

Supplementary Table 3. Potential secondary phenotypes of the genetic variants used for coffee

| SNP | Trait^1^ | *P* value |
| --- | --- | --- |
| rs2472297 | Creatinine in urine | 1.42e-25 |
|  | Leg fat percentage left | 3.25e-09 |
|  | Leg fat percentage right | 6.74e-09 |
|  | Impedance of arm right | 6.78e-09 |
|  | Potassium in urine | 1.59e-08 |
|  | Platelet distribution width | 2.44e-08 |
| rs66723169 | Body mass index | 2.96E-24 |
|  | Alcohol intake frequency | 1.03E-09 |
|  | Arm fat mass left | 4.25E-69 |
|  | Arm fat mass right | 6.47E-69 |
|  | Comparative body size at age 10 | 2.54E-65 |
|  | Comparative height size at age 10 | 9.10E-10 |
|  | Forced expiratory volume in 1-second, predicted | 2.78E-08 |
|  | Height | 3.32E-29 |
|  | Hip circumference | 5.73E-66 |
|  | Impedance of arm left | 2.17E-65 |
|  | Impedance of arm right | 6.40E-61 |
|  | Impedance of leg left | 5.95E-86 |
|  | Impedance of leg right | 5.02E-80 |
|  | Impedance of whole body | 9.84E-95 |
|  | Leg fat mass left | 1.26E-59 |
|  | Leg fat mass right | 5.17E-60 |
|  | Leg fat percentage left | 1.86E-23 |
|  | Leg fat percentage right | 7.32E-24 |
|  | Leg fat-free mass left | 3.58E-117 |
|  | Leg fat-free mass right | 6.96E-113 |
|  | Leg predicted mass left | 5.66E-117 |
|  | Leg predicted mass right | 3.34E-114 |
|  | Waist circumference | 1.31E-61 |
|  | Whole body water mass | 5.48E-124 |
|  | Worrier or anxious feelings | 6.84E-09 |
|  | Coronary artery disease | 3.96E-08 |
| rs574367 | Body mass index | 5.32E-29 |
|  | Childhood body mass index | 1.83E-13 |
|  | Body mass index in female non-smokers | 6.28E-21 |
|  | Body mass index in non-smokers | 3.15E-20 |
|  | Body mass index in non-smokers | 1.02E-23 |
|  | Body mass index in smokers | 1.20E-08 |
|  | Age at menarche | 3.61E-20 |
|  | Age at menarche | 7.00E-15 |
| rs1260326 | Granulocyte count | 6.81E-18 |
|  | Granulocyte percentage of myeloid white cells | 2.81E-27 |
|  | High light scatter percentage of red cells | 3.71E-22 |
|  | High light scatter reticulocyte count | 1.15E-18 |
|  | log eGFR creatinine | 3.40E-14 |
|  | Type II diabetes | 3.70E-09 |
|  | Height | 1.40E-11 |
|  | Total cholesterol | 3.08E-42 |
|  | Triglycerides | 2.29E-239 |
|  | 2 hour glucose | 2.26E-21 |
|  | Albumin | 4.36E-09 |
|  | C reactive protein | 5.40E-43 |
|  | FVII activity | 5.60E-25 |
|  | FVII in plasma | 6.20E-24 |
|  | Fasting blood glucose | 4.30E-13 |
|  | Fasting insulin | 2.74E-22 |
|  | Gamma glutamyl transferase | 3.90E-13 |
|  | HDL cholesterol mean size lipoprotein fraction concentration | 7.20E-10 |
|  | Hypertriglyceridemia | 6.50E-09 |
|  | IFT172 expression in Lymphocytes lymphoblastoid cell lines tissue | 7.00E-12 |
|  | IFT172 gene expression in adipose tissue | 1.50E-50 |
|  | Serum creatinine estimated glomerular filtration rate eGFR | 3.00E-14 |
|  | Serum urate | 5.90E-17 |
|  | Triglycerides | 5.60E-22 |
|  | Uric acid | 1.82E-09 |
|  | Alcohol consumption | 1.00E-21 |
|  | Cardiovascular disease risk factors | 2.00E-08 |
| rs10865548 | Body mass index females | 1.42E-08 |
|  | Body mass index males | 8.74E-10 |
|  | Body mass index | 1.86E-16 |
|  | Nonsyndromic striae distensae stretch marks | 2.88E-08 |
|  | Age at menarche | 2.12E-19 |
|  | Arm fat mass left | 1.27E-47 |
|  | Arm fat mass right | 7.04E-51 |
|  | Arm fat percentage left | 4.51E-31 |
|  | Arm fat percentage right | 2.71E-32 |
|  | Arm fat-free mass left | 1.10E-49 |
|  | Arm fat-free mass right | 1.79E-50 |
|  | Weight | 2.53E-61 |
|  | Whole body fat mass | 8.52E-43 |
|  | Whole body fat-free mass | 4.41E-55 |
|  | Whole body water mass | 1.69E-54 |
| rs4410790 | Habitual caffeine consumption | 2.36E-19 |
|  | Habitual caffeine consumption caffeinated coffee intake | 1.40E-29 |
|  | Habitual caffeine consumption female | 2.40E-16 |
|  | Habitual caffeine consumption never smokers | 1.80E-14 |
|  | Creatinine in urine | 1.32E-18 |
|  | Potassium in urine | 4.00E-09 |
|  | Sodium in urine | 6.52E-12 |
| rs34060476 | Granulocyte percentage of myeloid white cells | 1.03E-09 |
|  | High light scatter percentage of red cells | 1.70E-14 |
|  | High light scatter reticulocyte count | 1.86E-14 |
|  | Monocyte percentage of white cells | 1.84E-10 |
|  | Reticulocyte count | 9.75E-15 |
|  | Reticulocyte fraction of red cells | 2.28E-15 |
|  | Triglycerides | 9.73E-46 |
|  | Arm fat-free mass left | 1.16E-15 |
|  | Arm fat-free mass right | 1.32E-18 |
|  | Arm predicted mass left | 1.11E-16 |
|  | Arm predicted mass right | 8.67E-19 |
|  | Basal metabolic rate | 8.60E-14 |
|  | Hip circumference | 1.09E-08 |
|  | Impedance of arm left | 2.56E-18 |
|  | Impedance of arm right | 4.80E-19 |
|  | Impedance of leg right | 1.34E-09 |
|  | Impedance of whole body | 1.44E-15 |
|  | Leg fat-free mass left | 1.81E-10 |
|  | Leg fat-free mass right | 4.44E-12 |
|  | Leg predicted mass left | 1.47E-10 |
|  | Leg predicted mass right | 5.44E-12 |
|  | Self-reported gout | 2.91E-11 |
|  | Sitting height | 4.17E-17 |
|  | Sodium in urine | 6.41E-09 |
| rs1057868 | Mean platelet volume | 1.22E-09 |
|  | Creatinine in urine | 7.24E-10 |

Abbreviation: SNP, single nucleotide polymorphism; P-value: P-value for the genetic association；

^1^ Similar traits were only listed once.

Supplementary Table 4. Associations of single nucleotide polymorphisms for coffee consumption and renal cell carcinoma

| Exposure | Chr | SNP | Closest Gene | EA | EAF | Coffee consumption | | | Renal cell carcinoma in FinnGen | | | Renal cell carcinoma (men) in IARC | | | Renal cell carcinoma (women) in IARC | | |
| --- | --- | --- | --- | --- | --- | --- | --- | --- | --- | --- | --- | --- | --- | --- | --- | --- | --- |
|  |  |  |  |  |  | Beta | SE | *P* | Beta | SE | *P* | Beta | SE | *P* | Beta | SE | *P* |
| Coffee | 1 | rs574367 | *SEC16B* | T | 0.21 | 1.05 | 0.18 | 8.06E-09 | 0.1833 | 0.0599 | 0.5338 | 0.0729 | 0.0454 | 0.1088 | 0.0013 | 0.0579 | 0.9811 |
| Coffee | 2 | rs10865548 | *TMEM18* | G | 0.83 | 1.54 | 0.19 | 4.46E-15 | 0.8382 | 0.0626 | 0.6333 | -0.0239 | 0.0451 | 0.5962 | -0.05 | 0.0565 | 0.3757 |
| Coffee | 2 | rs1260326 | *GCKR* | C | 0.61 | 1.36 | 0.15 | 2.62E-19 | 0.6490 | 0.0486 | 0.1770 | -0.0195 | 0.0349 | 0.5753 | -0.0052 | 0.0439 | 0.9056 |
| Coffee | 7 | rs1057868 | *POR* | T | 0.29 | 1.97 | 0.16 | 5.26E-33 | 0.3952 | 0.0474 | 0.5120 | -0.0119 | 0.0382 | 0.7549 | 0.0964 | 0.0478 | 0.0439 |
| Coffee | 7 | rs34060476 | *MLXIPL* | G | 0.13 | 1.89 | 0.22 | 5.06E-18 | 0.1312 | 0.0683 | 0.3581 | -0.1144 | 0.058 | 0.0484 | -0.1382 | 0.0729 | 0.058 |
| Coffee | 7 | rs4410790 | *AHR* | C | 0.63 | 3.94 | 0.15 | 5.59E-141 | 0.6652 | 0.0492 | 0.2854 | -0.0426 | 0.0356 | 0.2317 | -0.0317 | 0.0439 | 0.4694 |
| Coffee | 7 | rs73073176 | *LOC101927630* | C | 0.87 | 2.31 | 0.22 | 5.56E-25 | 0.9119 | 0.0813 | 0.7556 | -0.0523 | 0.056 | 0.3504 | -0.0243 | 0.0697 | 0.7272 |
| Coffee | 11 | rs597045 | *OR8U8* | A | 0.69 | 1.07 | 0.16 | 6.62E-11 | 0.6402 | 0.0494 | 0.7107 | -0.0499 | 0.040 | 0.2123 | -0.0403 | 0.0497 | 0.4166 |
| Coffee | 14 | rs1956218 | *AKAP6* | G | 0.56 | 0.82 | 0.15 | 3.62E-08 | 0.4472 | 0.0468 | 0.8968 | -0.0271 | 0.0348 | 0.4359 | 0.0105 | 0.0432 | 0.8075 |
| Coffee | 15 | rs2472297 | *CYP1A1/2* | T | 0.27 | 4.54 | 0.17 | 5.19E-155 | 0.2477 | 0.0536 | 0.7795 | 0.0235 | 0.0439 | 0.5931 | 0.008 | 0.0532 | 0.8804 |
| Coffee | 18 | rs66723169 | *MC4R* | A | 0.23 | 1.47 | 0.18 | 9.88E-17 | 0.1731 | 0.0611 | 0.9759 | 0.0856 | 0.0419 | 0.0411 | 0.0192 | 0.0532 | 0.7177 |
| Coffee | 22 | rs2330783 | *SPECC1L-ADORA2A* | G | 0.99 | 4.53 | 0.63 | 1.57E-12 | 0.9928 | 0.2821 | 0.4739 | NA | NA | NA | NA | NA | NA |

Abbreviations: EA, effect allele; EAF, effect allele frequency; SE, standard error; SNP, single-nucleotide polymorphism; FinnGen, FinnGen Consortium; IACC, the International Agency for Research on Cancer.

Supplementary Table 5. Associations of single nucleotide polymorphisms for caffeine consumption and renal cell carcinoma

| Exposure | Chr | SNP | Closest Gene | EA | EAF | Caffeine consumption | | | Renal cell carcinoma in FinnGen | | | Renal cell carcinoma (men) in IARC | | | Renal cell carcinoma (women) in IARC | | |
| --- | --- | --- | --- | --- | --- | --- | --- | --- | --- | --- | --- | --- | --- | --- | --- | --- | --- |
|  |  |  |  |  |  | Beta | SE | P | Beta | SE | P | Beta | SE | P | Beta | SE | P |
| Caffeine | 7 | rs4410790 | AHR | C | 0.62 | 0.15 | 0.017 | 2.36E-19 | -0.0525 | 0.0492 | 0.2854 | -0.0427 | 0.0356 | 0.2317 | -0.0318 | 0.0439 | 0.4695 |
| Caffeine | 15 | rs2470893 | CYP1A1 | T | 0.31 | 0.12 | 0.016 | 5.15E-14 | 0.022 | 0.0517 | 0.6699 | 0.03649 | 0.0379 | 0.3367 | -0.0119 | 0.0469 | 0.7987 |

Abbreviations: EA, effect allele; EAF, effect allele frequency; SE, standard error; SNP, single-nucleotide polymorphism; FinnGen, FinnGen Consortium; IACC, the International Agency for Research on Cancer.

Supplementary Table 6. Estimated causal effect of coffee consumption on renal cell carcinoma with caffeine metabolite levels by inverse-variance weighted method

| Disease | No. of SNP | Association with caffeine metabolite levels | OR (95% CI) | P-value |
| --- | --- | --- | --- | --- |
| Renal cell carcinoma in FinnGen | 4 | low | 0.582(0.269,1.260) | 0.582 |
|  | 1 | high | 0.108(0.001,70.227) | 0.500 |
|  | 5 | unrelated | 2.316(0.213,25.148) | 0.490 |
| Renal cell carcinoma (male) in IARC | 4 | low | 0.809(0.445,1.468) | 0.485 |
|  | 5 | unrelated | 1.588(0.168,15.019) | 0.686 |
| Renal cell carcinoma (male) in IARC | 4 | low | 1.079(0.437,2.662) | 0.869 |
|  | 5 | unrelated | 0.606(0.091,4.061) | 0.606 |

Abbreviations:SNP: single-nucleotide polymorphism; OR: odds ratio; CI: confidence interval; FinnGen, FinnGen Consortium; IACC, the International Agency for Research on Cancer.
